# Preventing Overrides of Severe Drug Allergy Alerts Initiative: an Implemented System Upgrade

**DOI:** 10.1007/s10916-024-02116-5

**Published:** 2024-10-08

**Authors:** Laila Carolina Abu Esba, Samar Al Moaiseib, Norah Saud BinSabbar, Ghada Hussain Salamah Al Mardawi, Mufareh Alkatheri, Saleh Al Dekhail

**Affiliations:** 1 King Abdulaziz Medical City, Ministry of National Guard, Health Affairs, Joint Appointment Lecturer of Pharmacy Practice, King Saud Bin Abdulaziz University for Health Sciences, King Abdullah International Medical Research Centre, PO Box 22490, Riyadh, 11426 Kingdom of Saudi Arabia; 2 Corporate Clinical Information Management System, Information Technology, Riyadh, Saudi Arabia; 3 Saudi Medication Safety Center, MNGHA, P.O.Box 22490, 11426, Mail Code 2339 Riyadh, Saudi Arabia; 4 King Abdulaziz Medical City, Ministry of National Guard, Health Affairs, King Abdullah International Medical Research Centre, PO Box 22490, Riyadh, 11426 Kingdom of Saudi Arabia; 5 Department of Quality Improvement, King Abdulaziz Medical City, King Abdulaziz International Medical Research, Center, Ministry of National Guard - Health Affairs, Riyadh, Saudi Arabia; 6 Department of Medicine, King Abdulaziz Medical City, King Abdulaziz International Medical Research Center, Ministry of National Guard - Health Affairs, Riyadh, Saudi Arabia; 7 College of Medicine, King Saud Bin Abdulaziz, University of Health Sciences, Riyadh, Saudi Arabia; 8 King Abdulaziz Medical City, Ministry of NG-HA, KSAU-HS, P.O. Box 22490, Riyadh, 11426 Kingdom of Saudi Arabia

**Keywords:** Drug Hypersensitivity Reactions, Allergy Alert, Alert Fatigue, CPOE, Patient Safety, Drug-Allergy Reaction, Override

## Abstract

Administering medications to patients with documented drug hypersensitivity reactions (DHR) poses a significant risk for adverse events, ranging from mild reactions to life-threatening incidents. Electronic healthcare systems have revolutionized the modern clinical decision-making process, with built in warnings. However, as these alerts become a routine part of healthcare provider’s workflow, alert fatigue becomes a challenge. This study was conducted within the Ministry of National Guard Health Affairs (MNGHA), a government healthcare system in Saudi Arabia. A taskforce of experts was formed to develop an electronic path that would prevent unintentional overrides of severe drug allergy alerts. The system underwent rigorous testing, and monitoring parameters were established. We outline the implementation of a system upgrade designed to trigger an alternative interruption in the computerized physician order entry (CPOE) process, distinct from the regular allergy pop-up alerts. The alternate path is activated upon a CPOE with a drug-to-drug match and a documented severe drug allergy symptom, necessitating co-signature form another prescriber before proceeding. The adopted upgrade is a proactive approach to enhance medication safety in electronic healthcare systems, ensuring that serious allergy-related warnings are not overridden, ultimately enhancing patient safety. Further monitoring will confirm the safety and effectiveness of this measure. This study provides a model for institutions seeking to prevent allergy-related harm within their patient population.

## Introduction

Administering drugs to patients with known documented drug hypersensitivity reaction (DHR) to the same drug poses a significant risk for adverse events, which can range from mild reactions to life-threatening reactions. These types of events are, in theory, preventable when the risk is known, and avoiding the offending agent is typically recommended.

In a United States of America (USA) healthcare network study aimed at determining the prevalence of documented drug allergies in Electronic Health Records (EHRs), it was reported that (35.5%, *n* = 626 871) of patients had at least one reported drug allergy [1]. Similarly in a study conducted in Europe, a prevalence of (47%) was reported [2]. In a study in Saudi Arabia, immunological-type reactions were the most frequently reported adverse drug reactions (ADRs), accounting for (87%,* n* = 980/1156) of all spontaneously reported ADRs. Notably, over a six-year period, the only reported ADR resulting in a fatality case was a DHR [3].

In the USA, the estimated incidence of drug-induced anaphylaxis was 50–2000 episodes per 100,000 individuals, with antimicrobials being common culprits accounting for (26%) of anaphylaxis cases. Our institution’s reported rate of antimicrobial-associated anaphylaxis was 18.6 cases per 100,000 exposures. Therefore, the absolute risk of a drug anaphylaxis reaction is extremely low and even rarer are severe cutaneous adverse reaction (SCAR) [4–6].

Electronic healthcare systems and clinical decision support systems have revolutionized the modern clinical decision-making process for HCPs, offering tools and safeguards within the medication use processes. These systems aim to reduce a wide range of medication errors, including errors related drug allergies, drug-drug interactions and therapeutic duplications. Specifically, safeguards are built into these systems to issue alerts during the prescribing, dispensing, and administering phases when a medication to which the patient has a known allergy is being considered [7–9]. However, as these alerts become a routine part of healthcare provider’s workflow, alert fatigue becomes a challenge [2, 10–12].

Alert fatigue is a phenomenon where healthcare professionals become desensitized to the constant influx of alerts, warnings and notifications within the Computerized Physician Order Entry (CPOE) systems. In the context of medication allergies, alert fatigue can result in Health Care Providers (HCP) overriding allergy alerts, either out of habit or due to a perception that the risk is low. For medication allergy-related alerts, alert fatigue may be more problematic due to the multiple clinically insignificant cross-allergy alerts.

Luri et al. conducted a systematic review of drug allergy alert systems in hospital settings. They reported that drug allergy alerts were interruptive (i.e. pop-up messages) in all systems, triggered by a match at drug prescribing. Most systems (13 of 14 systems, 93%) allowed users to override the alert, and when overridden, entering a reason was mandatory. The study revealed a high override rate (97%), with subsequent adverse events as a result of overriding ranging from 0 to 6% [13].

Recently, there has been a growing emphasis and recommendations advocating proactive drug allergy delabeling, especially for antibiotics like penicillin [14]. This emphasis is well-justified, given the prevalence of inaccuracies in EHR labeling, poor allergy-history documentation, and the consequences of having to avoid entire drug classes, often resulting in the selection of less favorable drugs [2]. Nevertheless, while these efforts garner acknowledgment, it is crucial to recognize that the potential for severe, preventable drug allergic reactions remains a concern that cannot be ignored [15, 16].

The disparity between the extreme low absolute risk of severe DHRs and the prevalence of daily interruptive irrelevant alerts must be addressed. A probable solution proposed is to stratify the alerts and identify those with a high risk of severe events [17, 18]. Our manuscript explores a proactive approach to enhancing medication safety within electronic healthcare systems by introducing an innovative upgrade designed to prevent allergy-related harm. By adopting this enhancement, we aim to mitigate the risks associated with alert fatigue and ensure that serious allergy-related warnings are not overridden, ultimately enhancing patient safety.

In the subsequent sections, we describe the employed workflow for this system upgrade, outline the parameters for monitoring, and discuss the potential implications of our approach for healthcare organizations striving to improve medication safety. We believe this system enhancement serves as a model for institutions seeking to prevent allergy-related harm within their patient population.

## Methods

### Setting and Current State of the EHR

This patient safety initiative was conducted within the Ministry of National Guard Health Affairs (MNGHA), a government healthcare system in Saudi Arabia. MNGHA has been a pioneer in eHealth and was recognized with the Middle East Excellence Award in 2010 for its EHR implementation [19]. The organization employs 27,361 staff including 4,653 medical staff, 7,565 nursing staff, and 6,046 allied health staff. As of 2023, MNGHA comprises five medical cities, with a total of 103,743 inpatient admissions recorded in 2021. Additionally, it incorporates 71 Family Medicine and Primary Health Care clinics, collectively providing a bed capacity of 3,737 beds [20]. In 2016, the institution implemented a unified electronic medical record system, with the custom-designed system named “BESTCare health information management system”.

The medication safety program at MNGHA is a well-established service that operates under the umbrella of the quality and patient safety department. Its primary responsibility is to monitor and analyze all reported medication adverse events and subsequently recommend actions to prevent harm to patients. The institution also has an ADR committee, led by a clinical pharmacist.

### Allergy Information Within the EHRs

**Fig. 1:**
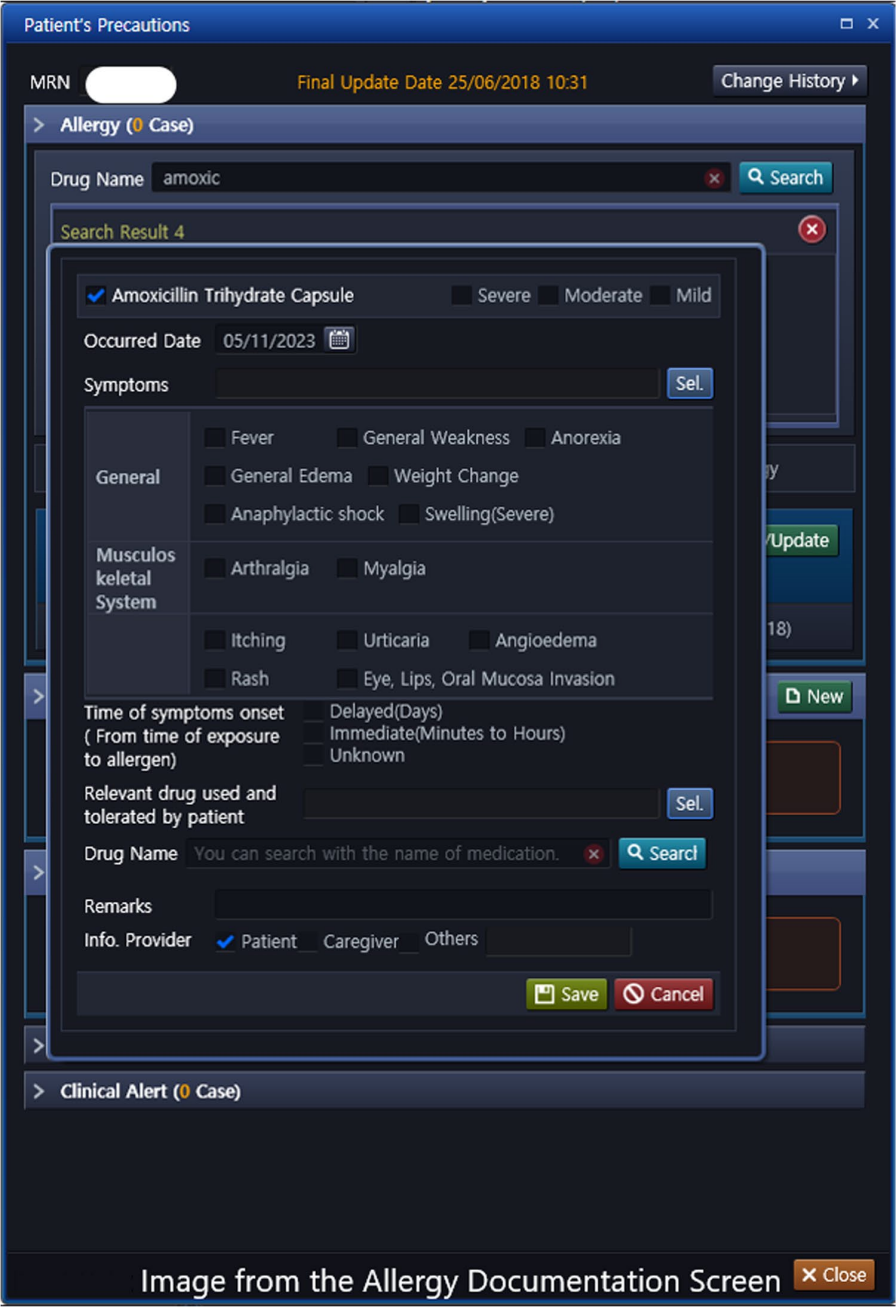
Image from the allergy documentation screen

At MNGHA, all patient’s drug allergies are documented by the attending physician in the EHR. The documentation requires the specific allergy information, as illustrated in Fig. 1. The allergy documentation consists of a look-up function with a comprehensive list of drug names, to match the entered text with a coded list of medications, medication classes, and ingredients. Furthermore, a set of the most commonly occurring signs and symptoms can be selected by clicking a checkbox, and free text may be entered if there is no coded match. Coded allergies are stored in the patient database as an ingredient or list of ingredients, signs and symptoms and time of onset are also coded. The information documented under this field of the EHR is extracted for all patients and displayed on an interactive dashboard that supports the ADR committee’s monitoring and analysis of ADRs [21].

**Fig. 2:**
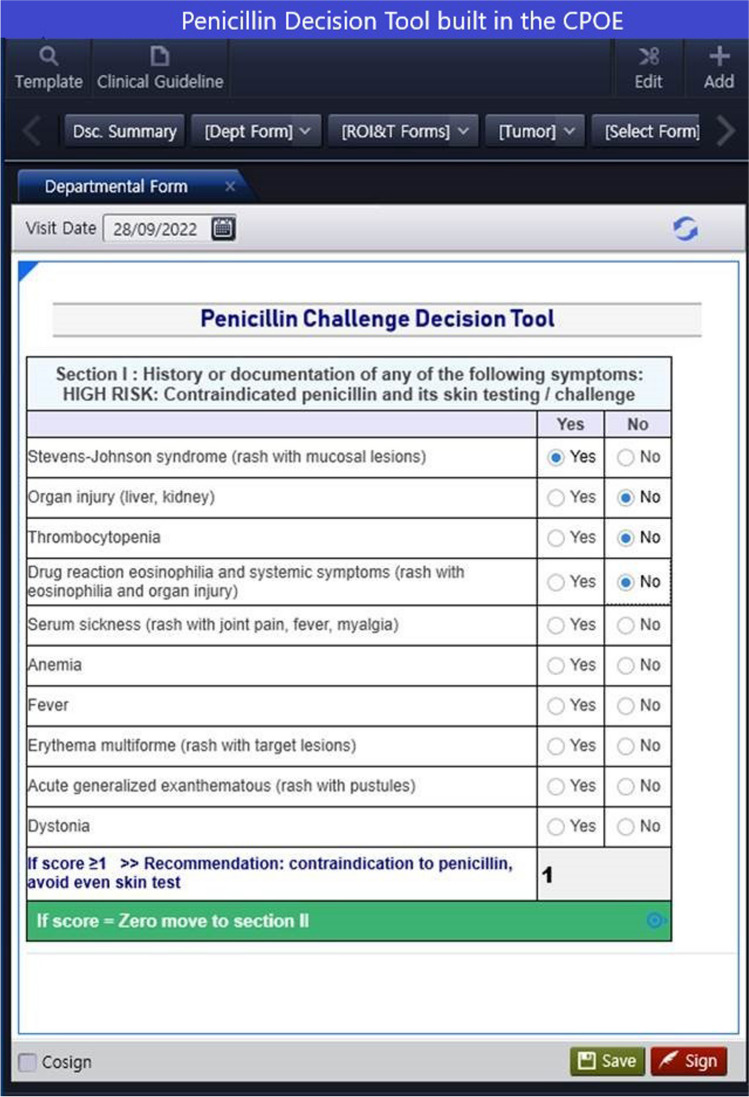
Penicillin decision tool built in the CPOE

The ADR committee at MNGHA has made progress over the past years in enhancing patient safety in the context of drug allergies through a series of projects aimed at improving allergy documentation and HCP awareness. The efforts have resulted in several key modifications within the EHRs, See Fig. 1:

- The introduction of a checkbox feature for common allergy symptoms to facilitate accurate documentation and ease coding, data reporting, and analysis. (The system no longer allows for free-text entry of allergen)
- The incorporation of a “time to onset of symptoms” field to differentiate between immediate and delayed types of reactions.
- The addition of a field to document relevant drugs that the patient can tolerate, to assist in decision-making (e.g. patient with a penicillin allergy but tolerated ceftriaxone)
- The inclusion of a free-text field for important comments, such as noting skin test results.
- An ongoing project to enable the uploading of photos of a patient’s cutaneous reaction.
- The development of an integrated penicillin allergy decision tool, which was developed based the review by Shenoy et al. [22] see Fig. 2.
- A continuing project focused on phasing out the older EHR system’s free-text documented allergies, which has been described by other institutions too [23].

#### Conceptual Context

The motivation for this project steamed from a critical incident, a serious medication error that resulted in patient harm, prompting the medication safety department to conduct a root-cause analysis. The root cause of the event was directly attributed to the overriding of an allergy alert (i.e. the prescriber, pharmacist, and nurse bypassed the allergy alert). Subsequently, a series of recommendations were devised to prevent harm and this project was conceptualized.

#### Task Force Formation

To address this pressing issue and implement the necessary changes, a multidisciplinary task force of pharmacists and physicians was assembled. This task force was composed of experts from the pharmacy, medication safety, allergy/immunology and the Clinical Information Management System (CIMS), in total six experts were in the team. Over a series of meetings continued until a comprehensive plan was developed and implemented, resulting in the described system upgrade below. Following extensive meetings with both end-users (prescribers) and task force members, a consensus was reached on a system upgrade with the features described in the results section.

## Results

**Fig. 3:**
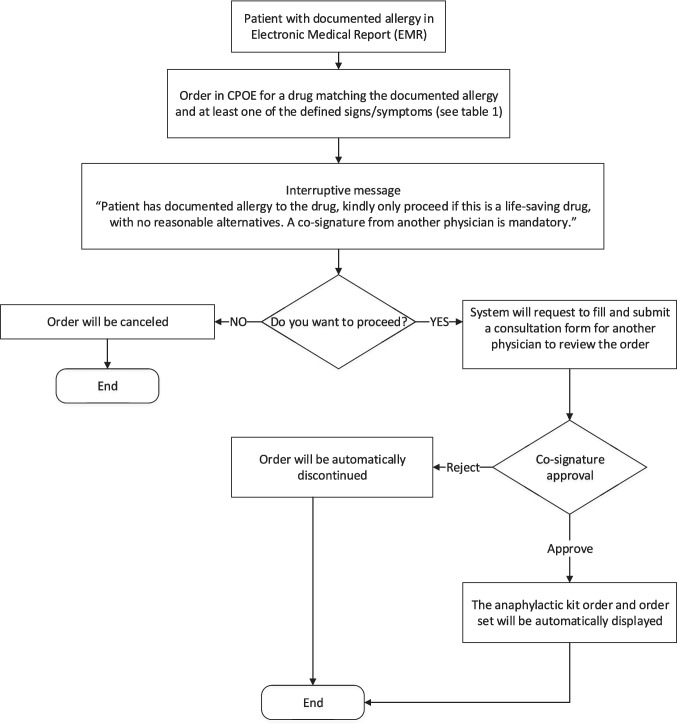
Workflow of the Allergy Upgrade in the System

**Fig. 4:**
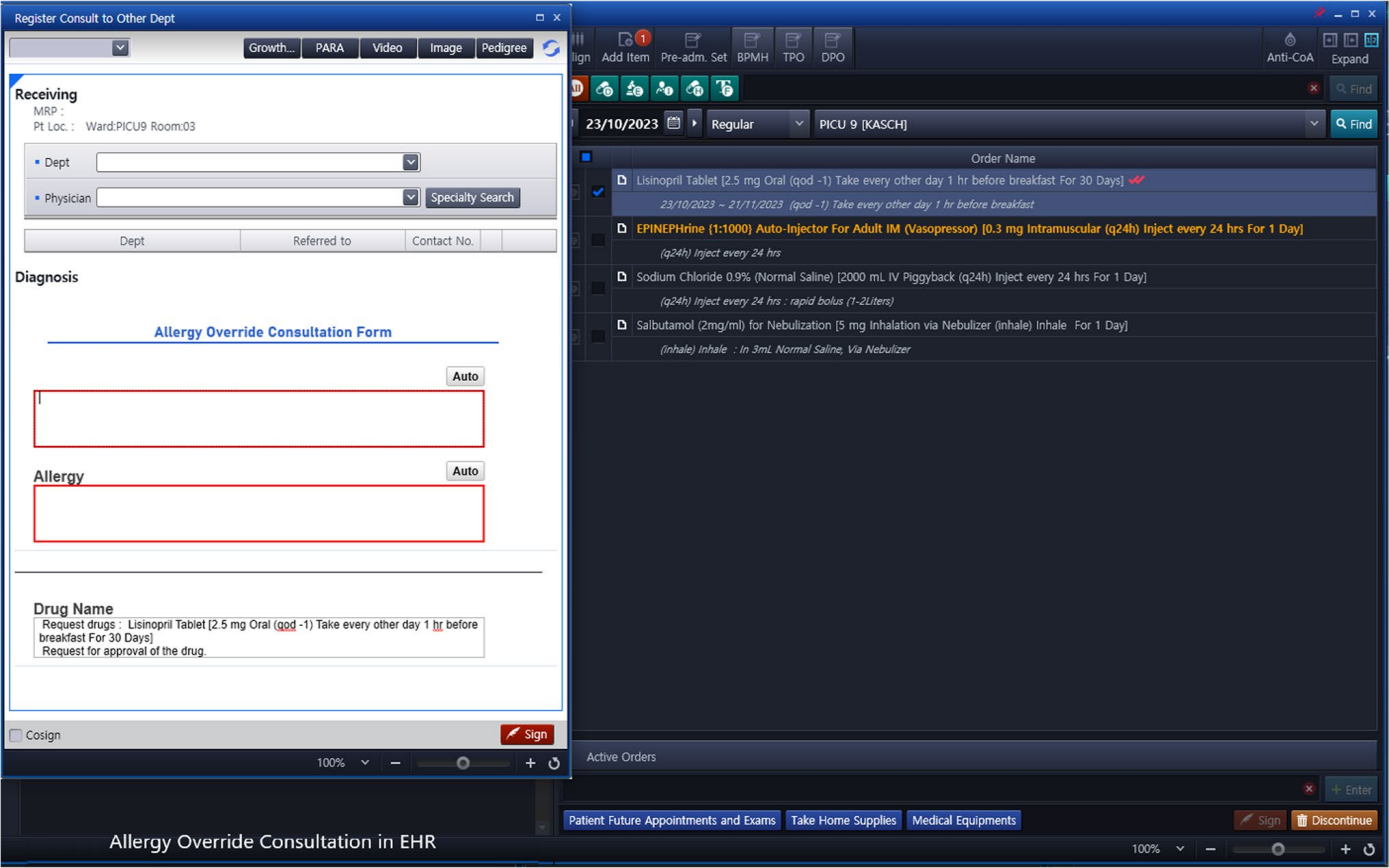
Allergy override consultation in EHR

**Fig. 5:**
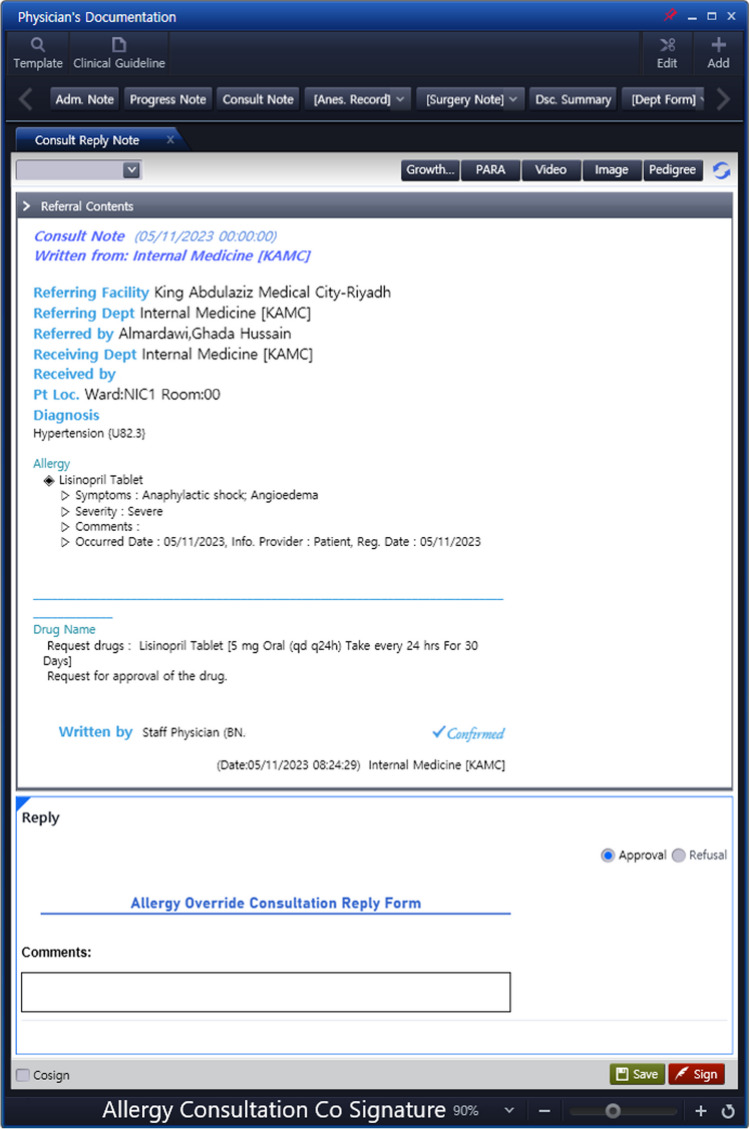
Allergy consultation co signature

**Fig. 6:**
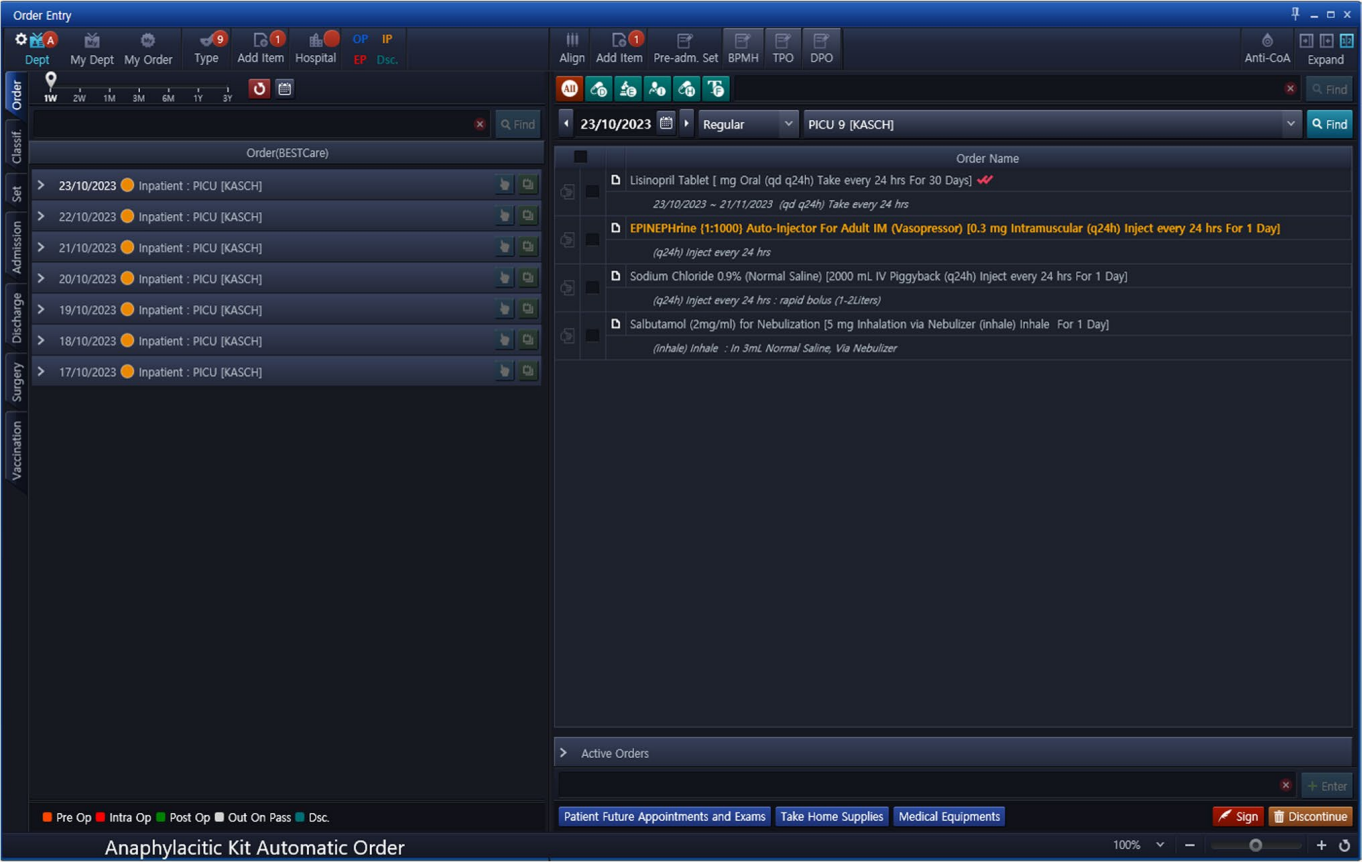
Anaphylacitic kit automatic order

**Fig. 7:**
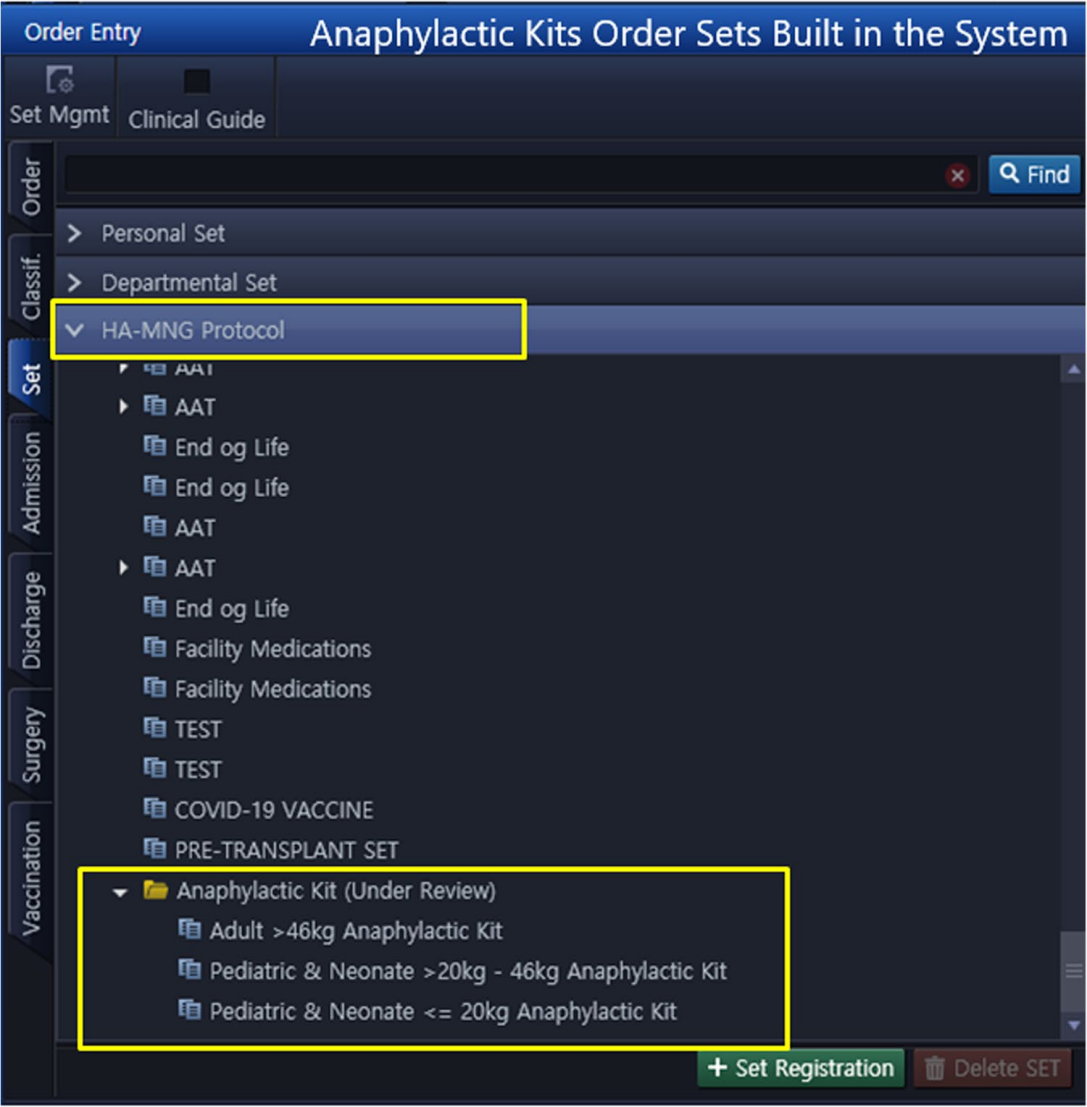
Anaphylactic kits order sets built in the system

System upgrade features:

1. Severe Terms List: A comprehensive list of specific signs, symptoms and medical terms that would signify a severe allergic reaction was compiled, mainly representing the common signs and symptoms of anaphylaxis and SCAR. See Table 1. This list was reviewed by allergy/immunology specialists and clinical pharmacists to ensure accuracy and relevance.
2. System Upgrade: Collaborative efforts and discussions led to the development of a criteria-based algorithm for allergy alerts. Unanimous agreement was reached among all members, and subsequent approvals were obtained from other concerned committees and higher administration. This upgrade selectively matches the prescription of a drug for a patient with a documented severe allergy to the drug, with at least one of the symptoms on the list (Table 1). When such match is found, it activates an electronic path that necessitates a co-signature from another prescriber before the order can proceed, or offers the option to cancel the order. Subsequently, the second prescriber has the authority to either approve or reject the order. For a detailed illustration of the workflow, see Fig. 3, and for images of the allergy consultation in the EHR, see Figs. 4 and 5.
3. Enhanced Safety Measures: To further ensure patient safety, when the order receives approval from a second prescriber, the system will automatically link this approval with an order for an anaphylactic kit with a built-in order set, ensuring that it is promptly available if required. See Figs. 6 and 7.

**Table 1:** The list of signs & symptoms and terms signifying a severe allergic reaction

Anaphylaxis
Swelling (Severe)
Pruritus with swelling of lips, tongue, uvula/palate
Tightness in the throat
Wheezing & cough
Bronchospasm
Shortness of breath
Cyanosis
Chest pain
Palpitation
Tachycardia
Bradycardia
Dysrhythmia
Fainting
Hypotension
Shock
Cardiac arrest
Hypoxemia
Desaturation
Reduced peak expiratory flow
Stridor
Collapse
Syncope
Loss of consciousness
Angioedema
Stevens–Johnson syndrome
Toxic epidermal necrolysis
Drug reaction with eosinophilia and systemic symptoms (DRESS)
Blistering rash

### Implementation

The upgraded system underwent rigorous testing and validation to ensure its accuracy and effectiveness in identifying severe allergy reactions. During the rollout, the CIMS team monitored for feedback and adjustments as needed. Finally in 2023 the piloting phase was completed and the upgrade was active.

### Members Discussion and Voting on Ingredient to Ingredient or Class/Group & Cross Reactions

Throughout the series of meetings, the issue of applying the new function to ingredient-to-ingredient match versus an ingredient-to-class and/or cross-reacting groups was thoroughly discussed. The task force members raised various points related to specificity and strength of evidence related to cross sensitivity, clinical significance, workflow efficiency and practicality. Following discussions, a formal vote was conducted, and consensus was reached. The decision was to implement the upgrade exclusively for ingredient-to-ingredient matches. Justification for this decision for some of the common cross sensitivity groups is detailed in Table 2 [24–27].

**Table 2:** Justification for the decision to implement the upgrade exclusively for ingredient-to-ingredient matches in relation to some of the common cross sensitivity groups

Cross reacting class	Rationale
NSAIDs and aspirin	There are primarily two distinct mechanisms underlying hypersensitivity reactions to NSAIDs & aspirin, one being a true allergy (IgE-mediated), and the other mediated by the inhibition of cox1 enzyme. It is important to note that symptoms of these reactions can overlap; making it challenging to differentiate between them without a detailed history of prior tolerance or intolerance to other NSAIDs, where in patients with cox1 inhibition mediated hypersensitivity would develop symptoms when exposed to any of the NSAIDs or aspirin. While in cases of true IgE-mediated allergies to NSAIDs, which are relatively uncommon, patients with an allergy to one NSAID may tolerate another NSAID, and the selection of an alternative NSAID can be based on avoiding those with a similar chemical structure For instance, if a patient exhibits a true allergy to diclofenac from the Heteroaryl acetic acids chemical group, choosing an NSAID like ibuprofen, which belongs to the Arylpropionic acids chemical group, may be a reasonable option [24]. It’s also important to consider the broader implications of restricting NSAID options for patients with a history of allergic reactions to one NSAID. Limiting prescribing to all NSAIDs without proper evaluation can lead to an increased risk of inappropriate opioid use and the associated potential for opioid dependence issues In addition, in cases of true NSAID allergy that is not mediated by cox1 inhibition, avoiding aspirin when clinically indicated is medically unacceptable
Opioids	Again, two distinct mechanisms are involved in opioid hypersensitivity. The more prevalent one involves pseudoallergies, which are associated with the immediate release of histamine. These pseudoallergies can sometimes be quite severe, mimicking an anaphylaxis. However, it’s important to note that they are technically anaphylactoid reactions. To manage these reactions, approaches such as slower infusion, administering smaller doses and opting for a synthetic opioids can effectively reduce these types of sensitivity reactions The other mechanism pertains to true allergies. In these cases, similarities in chemical structures and source of opioids (natural vs synthetic) is used to guide selection rather than avoiding the whole opioid class, which can be unreasonable when dealing with severe pain [25].
Cephalosprins and penicillin cross allergy	It is widely accepted now that the risk of cross-sensitivity between beta-lactam antibiotics and cephalosporins is minimal. Consequently, the selection of antibiotics is based on the similarity of the R side chains of beta lactams, rather than a complete avoidance of the entire cephalosporin group. In addition, the use of third or fourth generation cephalosporins is considered acceptable, even when a patient has a history of anaphylaxis to penicillin. This approach plays a crucial role in mitigating antimicrobial resistance and avoidance of less favorable and possibly more toxic antibiotics [26].
Sulfonamide drugs	Evidence suggests that cross-allergy between sulfonamide antibiotics and sulfonamide non-antibiotics is largely theoretical and lacks support from observational studies to be of clinical significance. Within the sulfonamide antibiotic class, sulfamethoxazole/trimethoprim is the only drug currently and very commonly used in practice, while the list of non-antibiotics sulfonamides is exhaustive [27]. Therefore, implementing a strict avoidance of non-antibiotics sulfonamide in patients with a history of allergy to sulfamethoxazole/trimethoprim is unpractical and would lead to significant unnecessary interruptions

*Cox1* Cyclooxygenase-1; *NSAID* Nonsteroidal anti-inflammatory drugs

### Monitoring Parameters

The team agreed on monitoring the following aspects to determine the safety and effectiveness of the system upgrade:

- User survey: a sample of prescribers will be surveyed to ask for feedback and satisfaction with the new upgrade
- CIMS will provide the team with a report on the number of alerts being triggered in the system, the number of times the orders were canceled, and the number of times a co-signature was forwarded and the rate of approval and rejection of the co-signer.
- Appropriateness of order cancelation and clinical consequences (assessed by a clinical pharmacists and an allergist)
- Appropriateness of orders approved and continued (assessed by a clinical pharmacists and an allergist)

The duration of monitoring was agreed to continue for two years due to the low incidence rate of severe allergic reactions meeting the criteria. The results will be published once completed.

## Discussion

The Institute for Safe Medication Practice (ISMP) has developed a medication safety self-assessment tool for hospitals, which states two pivotal key elements crucial for ensuring the safe use of medications related to drug allergies. The first element emphasizes the importance of CPOE systems’ capability to detect drugs to which patients may be allergic, including cross allergies, and provide a clear warning during order entry, and require practitioners to enter an explanation to override the warning. However, another key element highlights the necessity for CPOE systems to incorporate a tiered severity rating for allergies, based on the patient’s reaction to the drug. This tiered approach aims to limit alert fatigue from drug intolerances that are not true allergies, recognizing the importance of limiting clinically irrelevant drug allergy alert interruptions [18].

Our study introduces a novel system upgrade, which addresses a critical aspect of patient safety within healthcare systems, preventing severe allergic reactions. We describe the implementation of a system upgrade designed to prevent the overriding of severe drug allergy alerts. This intervention comes as a pressing need for ways to mitigate consequences of alert fatigue prevalent in electronic healthcare systems [28].

Many healthcare institutions have recognized the significant impact of inaccurate drug labeling in EHRs on patient care and have undertaken substantial projects to rectify and optimize EHR functionality. For instance, Li et al. undertook an initiative aimed at systematically reducing free-text allergy entries and transferring them into coded entries within the EHR allergy module [23]. A similar initiative was undertaken at our institution during the transition from the old EHR to the new EHR (BESTCare). Additionally, other institutions have developed tools to assist in appropriate drug allergy labeling. Varghese et al. for example, developed a Natural Language Processing (NLP) tool that analyzes and interprets drug challenge test results, compares the result against EHR allergy lists to detect potential discrepancies in allergy documentation [26]. However, a notable gap in the literature is the absence of published experiences related to EHR system changes exclusively targeting identification of patients at high risk of severe DHR and routing them through a separate path, distinct from the traditional pop-up alerts.

While institutions continue to explore strategies to reduce the burden of alert fatigue and minimize insignificant and clinically irrelevant allergy alert interruptions, together with ongoing allergy de-labeling efforts, our approach presents an effective means to reduce the risks associated with the unintentional overriding of severe and relevant patient allergy alerts. This initiative has the potential to significantly enhance patient safety by reducing the occurrence of severe allergic reactions to medications.

The importance of our experience lies in its execution within a well-established, multisite, and large healthcare system known for its pioneering in eHealth. Nonetheless, it is important to acknowledge certain limitations in our approach. Allergies with severe symptoms not included in our predefined list will bypass the system’s intervention and will only trigger the regular allergy pop-up alert. Additionally, while the symptoms on the list are indicative of severe allergic reactions, there may be clinical instances where drug avoidance is neither clinically suitable nor necessary. It is also crucial to note that the accuracy of the allergy documentation and the details of the symptoms and onset would be important for this measure to be successful is preventing harm.

Finally, with updates in practice focusing on de-labeling allergies, drug challenge and deemphasizing the role of skin testing we believe it is an important initial step to prevent harm from severe and serious allergic reactions [29, 30].

## Conclusion

The system upgrade described in this paper comes as a continuation of efforts to ensure that no patient suffers a preventable severe allergic drug reaction as a result of alert fatigue, further monitoring will be conducted and reported to confirm the safety and effectiveness of this measure. While continued efforts are explored to safely eliminate irrelevant allergy alerts.

## Data Availability

No datasets were generated or analysed during the current study.
